# Supplementary material for: A Humanized Yeast Phenomic Model of Deoxycytidine Kinase to Predict Genetic Buffering of Nucleoside Analog Cytotoxicity
Source: Genes (Basel). 2019 Sep 30;10(10):770. doi: 10.3390/genes10100770 (PMC6826991; doi:10.3390/genes10100770)

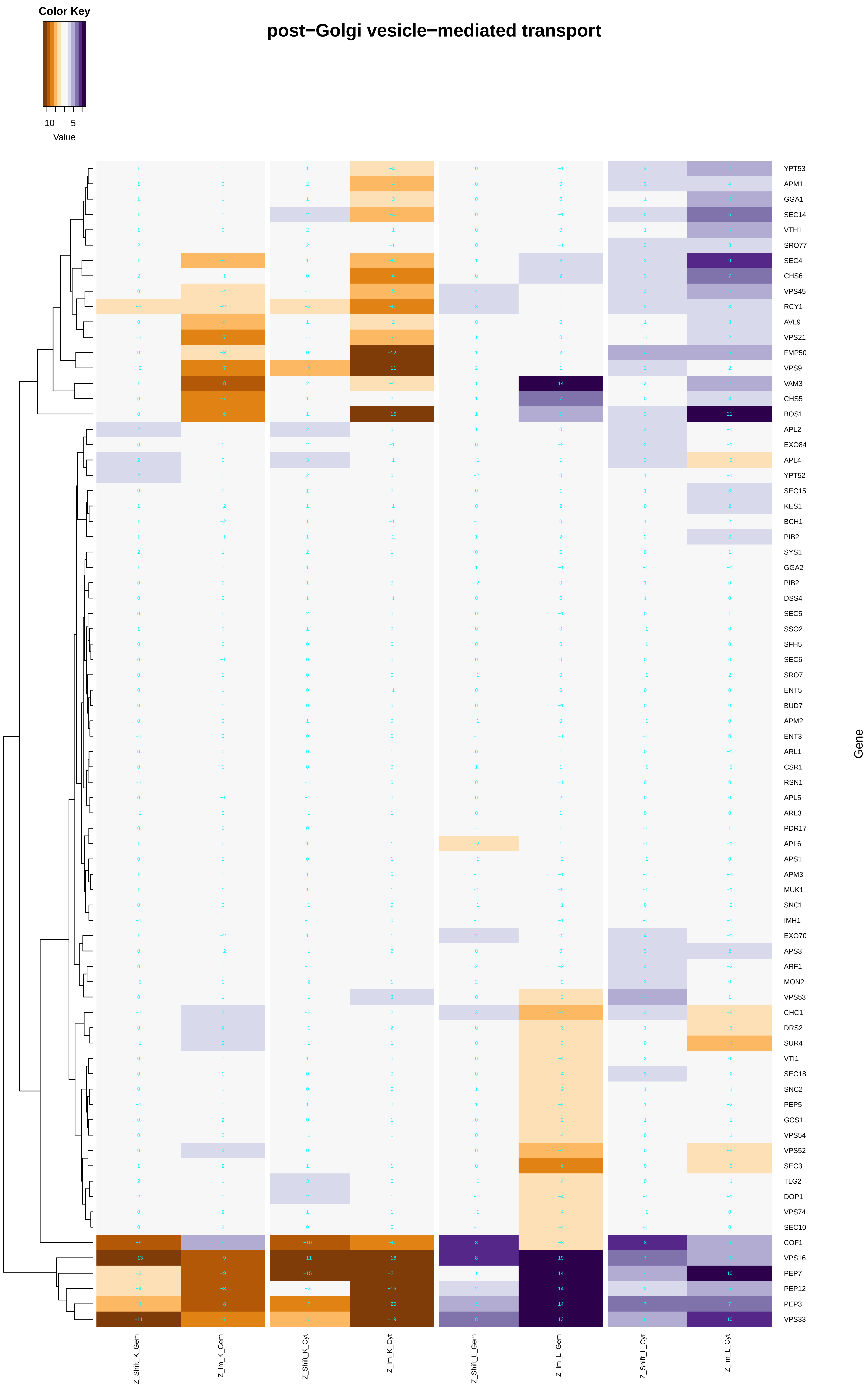
Color Key
−10 5
Value
post−Golgi vesicle−mediated transport
YPT53
APM1
GGA1
SEC14
VTH1
SRO77
SEC4
CHS6
VPS45
RCY1
AVL9
VPS21
FMP50
VPS9
VAM3
CHS5
BOS1
APL2
EXO84
APL4
YPT52
SEC15
KES1
BCH1
PIB2
SYS1
GGA2
PIB2
DSS4
SEC5
SSO2
SFH5
SEC6
SRO7
ENT5
BUD7
APM2
ENT3
ARL1
CSR1
RSN1
APL5
ARL3
PDR17
APL6
APS1
APM3
MUK1
SNC1
IMH1
EXO70
APS3
ARF1
MON2
VPS53
CHC1
DRS2
SUR4
VTI1
SEC18
SNC2
PEP5
GCS1
VPS54
VPS52
SEC3
TLG2
DOP1
VPS74
SEC10
COF1
VPS16
PEP7
PEP12
PEP3
VPS33
Gene
Z_Shift_K_Gem
Z_Im_K_Gem
Z_Shift_K_Cyt
Z_Im_K_Cyt
Z_Shift_L_Gem
Z_Im_L_Gem
Z_Shift_L_Cyt
Z_Im_L_Cyt

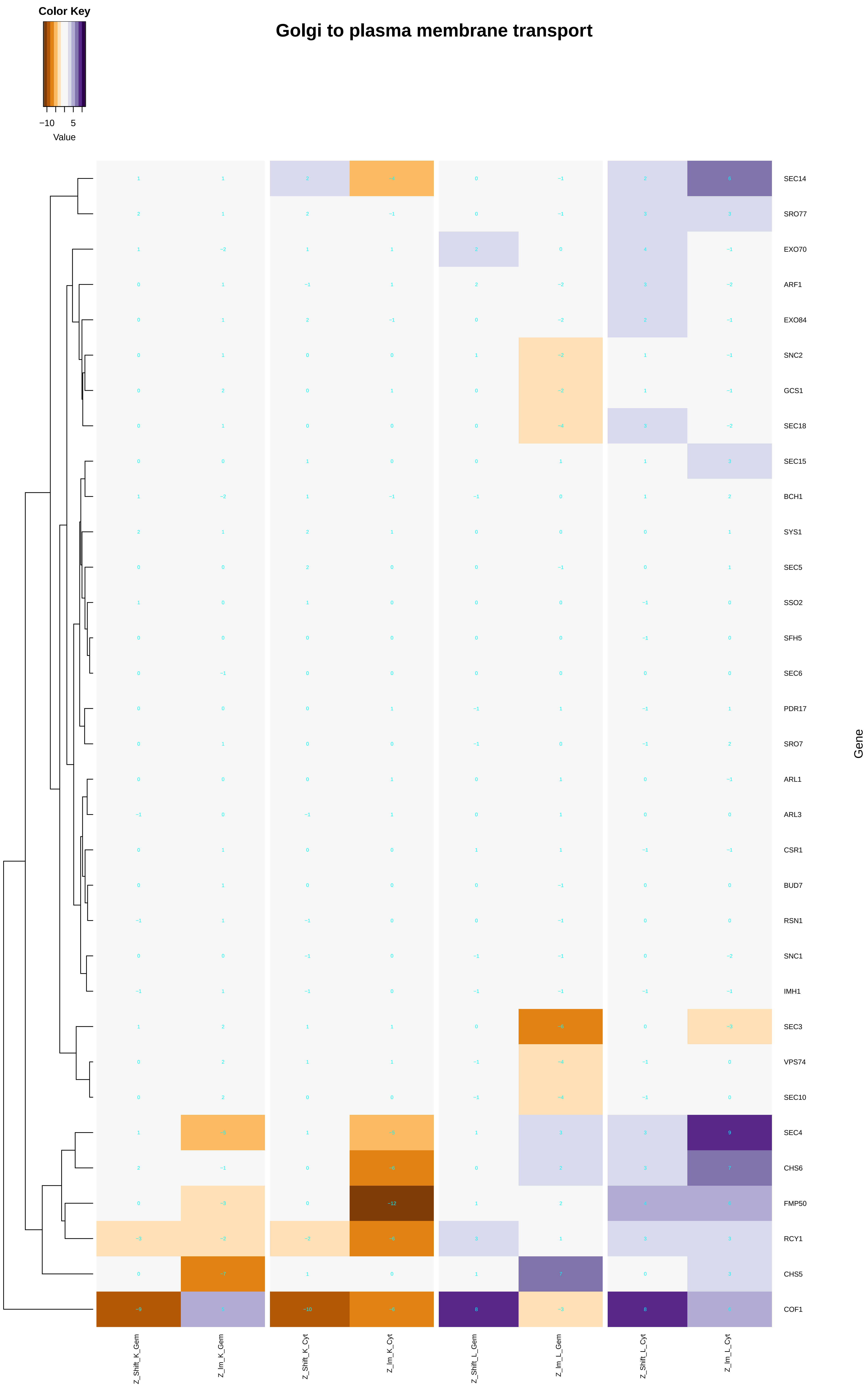
Color Key
−10
5
Value
Golgi to plasma membrane transport
SEC14
SRO77
EXO70
ARF1
EXO84
SNC2
GCS1
SEC18
SEC15
BCH1
SYS1
SEC5
SSO2
SFH5
SEC6
PDR17
SRO7
ARL1
ARL3
CSR1
BUD7
RSN1
SNC1
IMH1
SEC3
VPS74
SEC10
SEC4
CHS6
FMP50
RCY1
CHS5
COF1
Gene
Z_Shift_K_Gem
Z_Im_K_Gem
Z_Shift_K_Cyt
Z_Im_K_Cyt
Z_Shift_L_Gem
Z_Im_L_Gem
Z_Shift_L_Cyt
Z_Im_L_Cyt

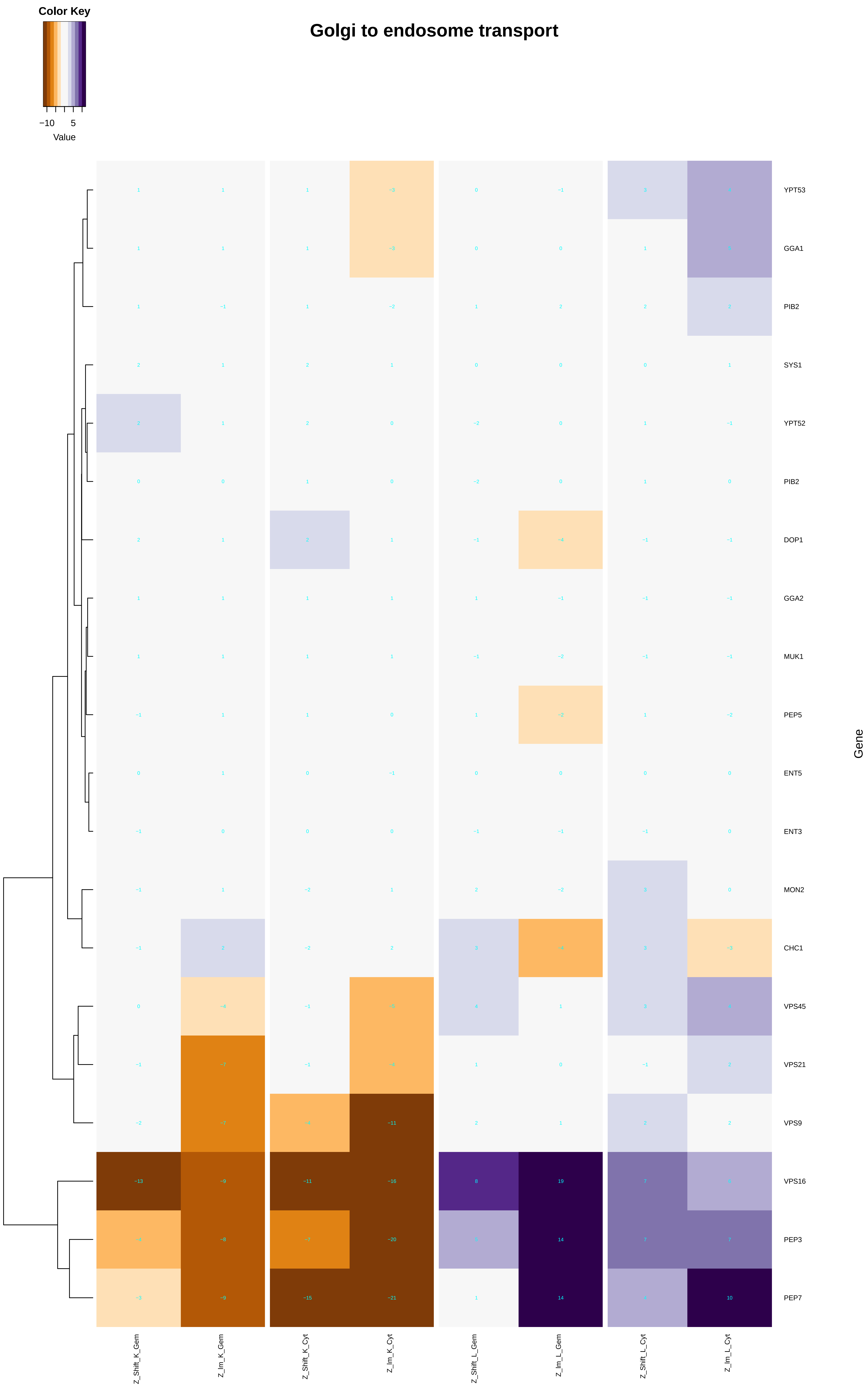
Golgi to endosome transport
Color Key
−10
5
Value
YPT53
GGA1
PIB2
SYS1
YPT52
PIB2
DOP1
GGA2
MUK1
PEP5
ENT5
ENT3
MON2
CHC1
VPS45
VPS21
VPS9
VPS16
PEP3
PEP7
Gene
Z_Shift_K_Gem
Z_Im_K_Gem
Z_Shift_K_Cyt
Z_Im_K_Cyt
Z_Shift_L_Gem
Z_Im_L_Gem
Z_Shift_L_Cyt
Z_Im_L_Cyt

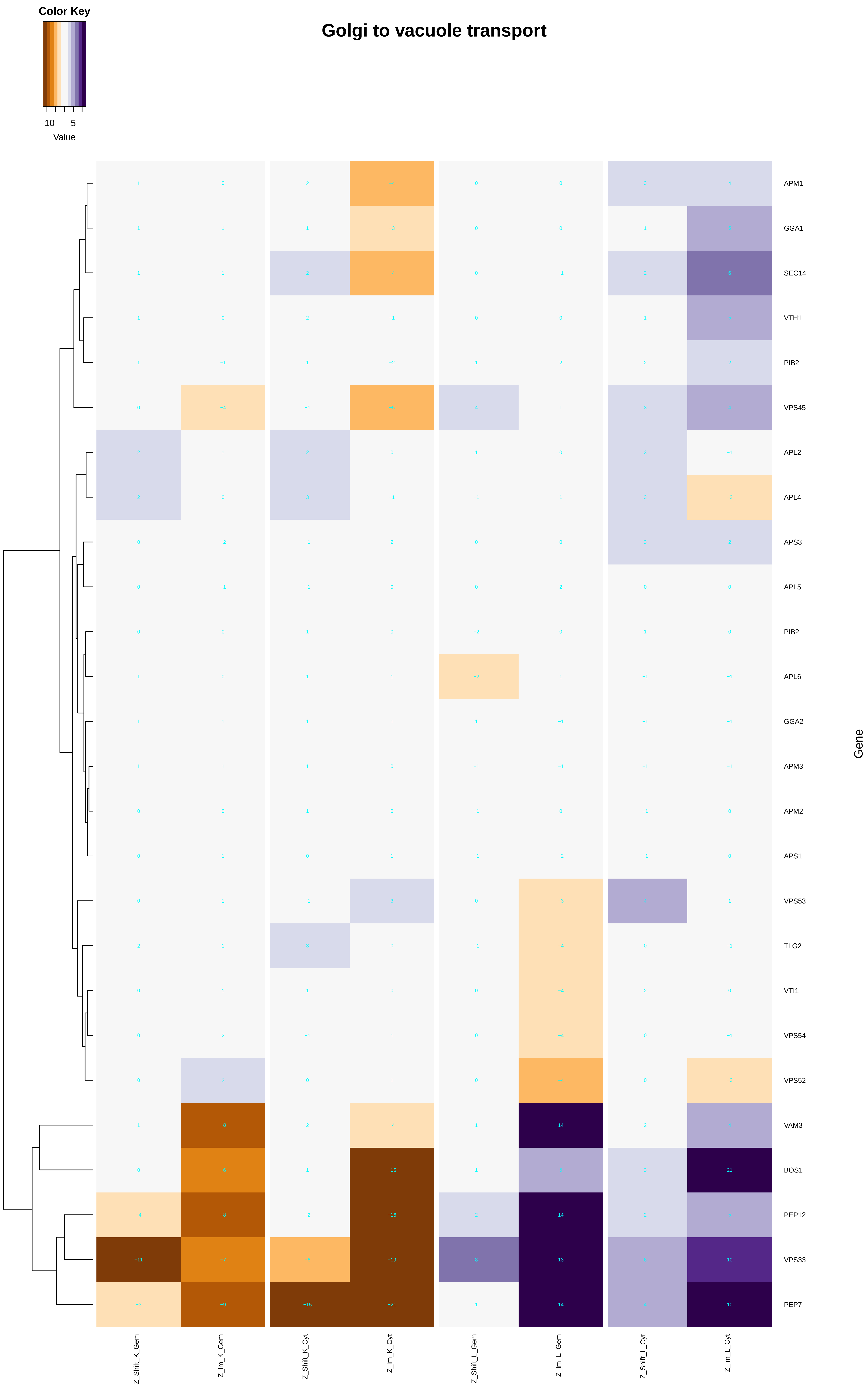
Color Key
−10
5
Value
Golgi to vacuole transport
APM1
GGA1
SEC14
VTH1
PIB2
VPS45
APL2
APL4
APS3
APL5
PIB2
APL6
GGA2
APM3
APM2
APS1
VPS53
TLG2
VTI1
VPS54
VPS52
VAM3
BOS1
PEP12
VPS33
PEP7
Gene
Z_Shift_K_Gem
Z_Im_K_Gem
Z_Shift_K_Cyt
Z_Im_K_Cyt
Z_Shift_L_Gem
Z_Im_L_Gem
Z_Shift_L_Cyt
Z_Im_L_Cyt

# Golgi to plasma membrane protein transport

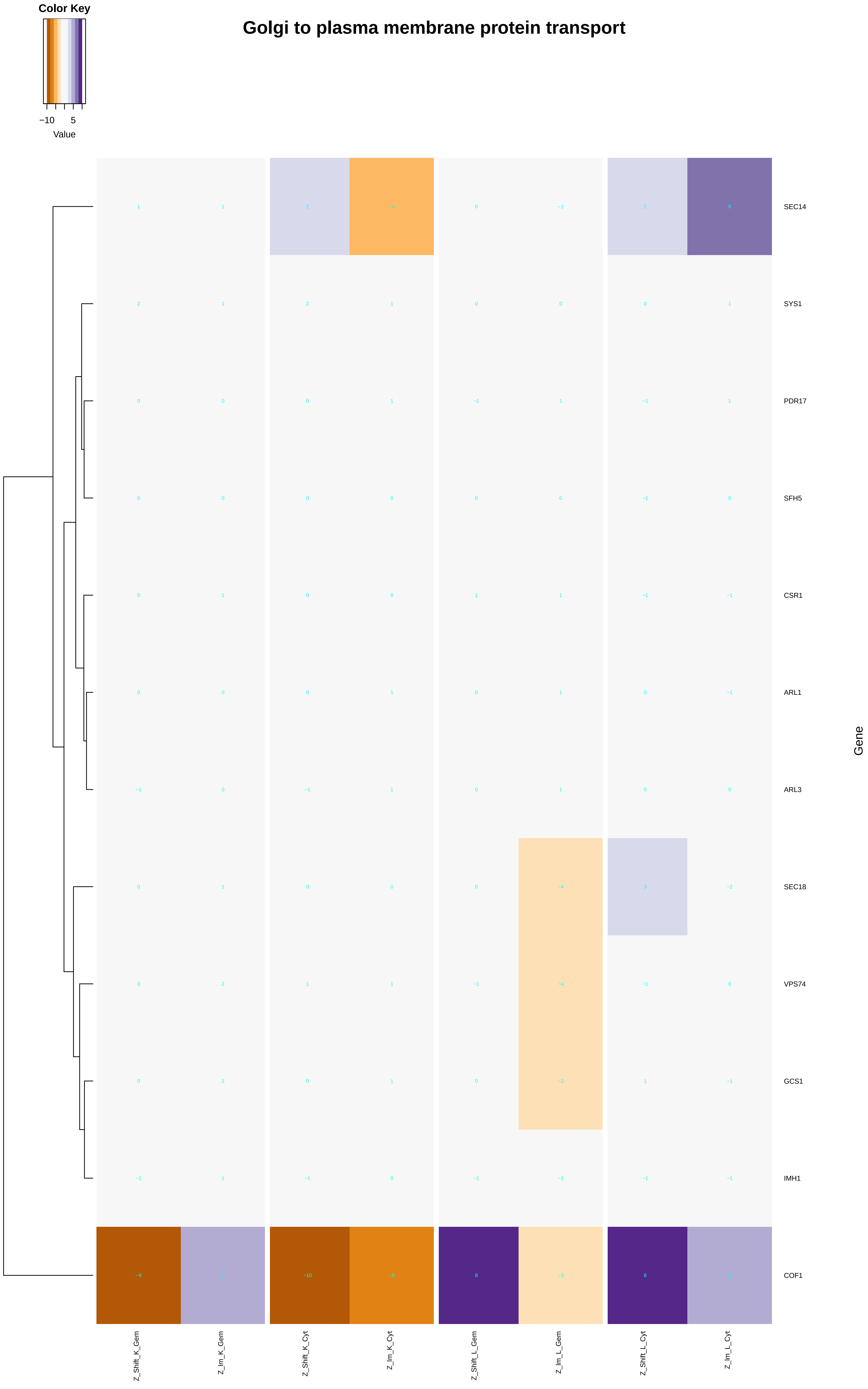

Supplement: Supplementary file 1 [file genes-10-00770-s001.zip › Additional_Files/Additional_File7_GOTermHeatmaps.zip!/Additional_File7_GOTermHeatmaps/post-Golgi_vesicle-mediated_transport.pdf]
